# Thymol combined with SAEW for the eradication of mature *Pseudomonas aeruginosa* biofilms and reduction of bacterial virulence

**DOI:** 10.3389/fmicb.2025.1547632

**Published:** 2025-06-27

**Authors:** Zhexiao Ma, Changrui Qian, Zeyong Zhong, Zhuocheng Yao, Congcong You, Jianming Cao, Cui Zhou, Jianzhong Ye

**Affiliations:** ^1^Department of Clinical Laboratory, The First Affiliated Hospital of Wenzhou Medical University, Key Laboratory of Clinical Laboratory Diagnosis and Translational Research of Zhejiang Province, Wenzhou, China; ^2^School of Laboratory Medicine and Life Science, Wenzhou Medical University, Wenzhou, China

**Keywords:** *Pseudomonas aeruginosa*, SAEW, thymol, anti-biofilm, antitoxicity

## Abstract

**Introduction:**

Biofilms formed by *Pseudomonas aeruginosa* (*P. aeruginosa*) are a major challenge in clinical settings due to their resilience and contribution to persistent infections, especially in patients with indwelling medical devices. There is an urgent need for effective strategies to disrupt mature biofilms and control associated infections.

**Methods:**

This study investigated the combined antibacterial activity and mature biofilm eradication efficacy of slightly acidic electrolyzed water (SAEW) and thymol against *P. aeruginosa* PAO1 through mature biofilm removal assays. The underlying antibacterial mechanism was explored by measuring intracellular reactive oxygen species (ROS) levels. The impact of the combined treatment on the expression of PAO1 virulence genes was assessed using RT-qPCR. Additionally, the safety of the combination was evaluated through acute dermal toxicity and ocular irritation tests in mice.

**Results:**

The combination of thymol and SAEW effectively disrupted mature biofilms, significantly reduced bacterial load on medical catheters, and enhanced ROS production. Furthermore, the treatment downregulated key virulence genes, *lasA* and *lasB*, which are critical for elastin degradation and pathogenicity. Safety assessments confirmed no acute skin or ocular toxicity, indicating its suitability for clinical applications.

**Discussion:**

Thymol-enhanced SAEW shows great potential as a safe and effective strategy for biofilm eradication and infection control, paving the way for innovative approaches to combat antimicrobial-resistant pathogens in healthcare settings.

## 1 Introduction

Given the potential environmental hazards associated with residues of traditional disinfectants, slightly acidic electrolyzed water (SAEW) has emerged as a novel, safe, and environmentally friendly alternative (Kurahashi et al., 2021). SAEW, also known as hypochlorous acid water, is produced by electrolyzing a dilute electrolyte (typically containing NaCl and/or HCl) in a non-membrane electrolytic cell. The pH of SAEW is close to neutral (5.0–6.5), and the effective chlorine is almost entirely present in the form of hypochlorous acid molecules, allowing for rapid eradication of various bacteria and fungi, such as *Escherichia coli, Listeria monocytogenes, Rhizopus stolonifer*, etc. (Luo and Oh, 2016; Ye et al., 2017; Li L. et al., 2021). Compared to other commonly used disinfectants, such as sodium hypochlorite and 75% ethanol, SAEW does not leave disinfectant residues, does not corrode instrument surfaces, and is non-irritating to the eyes, skin, and respiratory tract (Zang et al., 2019). Due to its broad-spectrum antimicrobial properties, instantaneous high efficacy, low cost, and environmental safety, SAEW has a wide range of applications in cleaning and disinfection across fields such as food, healthcare, environment, and surfaces (Cao et al., 2009; Ni et al., 2015).

In recent years, the potential hazards associated with the improper use of chemical disinfectants have garnered increasing attention. Existing studies indicate that the use of chemical disinfectants can induce the emergence of disinfectant-resistant bacteria, some of which also exhibit cross-resistance to clinical antimicrobial agents (Bland et al., 2021; Yeung et al., 2022). In addition, due to the disadvantages of SAEW being easily decomposed and volatile when heated, and not suitable for long-term storage, there is an urgent need for combination therapy to achieve short-term rapid killing of bacteria and long-term reduction of bacterial toxicity. Similar to other chlorine-containing disinfectants, SAEW exerts its antimicrobial effect through the oxidative potential of hypochlorous acid. However, its potential to contribute to bacterial resistance and enhance virulence may pose limitations to its broader application. Additionally, the emergence of strains resistant to chlorine-containing disinfectants could further restrict the antibacterial efficacy of SAEW (Russell, 1986). Therefore, there have been studies that enhance the antibacterial effect by combining SAEW with other compounds, such as didecyldimethylammonium bromide and fumaric acid (Tango et al., 2014; Li et al., 2022). However, there is still a lack of research on the combined application of SAEW and plant derived quorum sensing inhibitors (QSIs).

QSIs do not directly kill bacteria but target the quorum sensing (QS) system, interfering with the formation of biofilms and the production of virulence factors, thereby effectively preventing the development of bacterial resistance (Deryabin et al., 2019). Thymol, a natural phenolic compound (2-isopropyl-5-methylphenol) derived from various plants such as oregano (*Origanum vulgare*) and thyme (*Thymus vulgaris*), is a well-established QSI. Its properties have gained significant attention, particularly for its ability to disrupt bacterial cell membranes and inhibit biofilm formation (Marchese et al., 2016; Walczak et al., 2021; Zhu et al., 2021; Saptami et al., 2022). The combination of plant-derived QSI and SAEW is expected to overcome the risks associated with traditional chemical disinfectants, such as inducing bacterial resistance and enhancing pathogenicity, making it an ideal choice for safe and effective disinfection.

In summary, this study aimed to investigate the combined effect of thymol and SAEW on the clearance of mature *Pseudomonas aeruginosa* (*P. aeruginosa*) biofilm, which often forms on host tissues and medical device surfaces and hinders antibacterial treatment (Jeong et al., 2024). This study focused on the antibacterial mechanism of the combination of SAEW and thymol and its potential impact on the expression of virulence genes. By elucidating the role of thymol in enhancing the antimicrobial and antibiofilm effects of SAEW, we strive to promote the development of effective and safe biofilm management strategies in clinical applications.

## 2 Materials and methods

### 2.1 Bacterial isolates

This study selected the model strain *P. aeruginosa* PAO1 (ATCC15692) as the research subject. The strain was preserved at −80°C in Luria-Bertani (LB) broth supplemented with 30% glycerol for future studies.

### 2.2 Antimicrobial susceptibility testing

We utilized the microbroth dilution method to determine the minimum inhibitory concentrations (MICs) of thymol for the PAO1 strain, assessing its susceptibility to the treatment (Humphries et al., 2021). The procedure was conducted based on previously established protocols with minor modifications. Briefly, 96-well plates were prepared with cation-adjusted Mueller-Hinton broth (CAMHB) and a series of drug concentrations created through geometric dilutions. Subsequently, 100 μL of a bacterial suspension (1 × 10^6^ CFU/mL) was added to each well, and the plates were incubated at 37°C for 16–18 h. The MIC was defined as the lowest drug concentration that completely inhibited visible bacterial growth.

### 2.3 Preparation of slightly acidic electrolyzed water

In this study, SAEW was prepared using an SAEW generator (SHC-50MS SAEW generator, Shandong Shenghuai Bioengineering Co., Ltd.). To produce an 80 ppm SAEW solution, 5 g of NaCl was dissolved in 1,600 mL of ddH_2_O and allowed to sit for 30 s before being introduced into the generator for electrolysis. The resulting 80 ppm SAEW was then diluted with ddH_2_O to achieve the required concentrations for the experiments. The pH of the SAEW was determined using a pH meter (Yan et al., 2021). For subsequent experiments, a concentration of 30 ppm SAEW was selected, as it represents a commonly used concentration in practical applications and is also the standard experimental concentration adopted by most SAEW antimicrobial research studies (Kim et al., 2018; Li et al., 2022; Yan et al., 2022).

### 2.4 Eradication of mature biofilms formed by *P. aeruginosa* PAO1

Due to the unstable physical and chemical properties of SAEW, this study optimized the experimental procedure to ensure its effectiveness in eradicating mature *P. aeruginosa* biofilms (Yan et al., 2022). Thymol (purity ≥98.5%) used in this study was purchased from Sigma-Aldrich. The experimental workflow was as follows: First, *P. aeruginosa* was cultured overnight until it reached the logarithmic growth phase. The bacterial suspension was then diluted to 1 × 10^6^ CFU/mL, and 1 mL of the suspension was added to a 24-well plate. The plate was then incubated statically at 37°C for 24 h to allow the formation of a mature biofilm. Before drug treatment, the old culture medium was discarded, and the biofilm was rinsed with 1 × PBS to remove planktonic bacteria. Once the mature biofilms were established, monotherapy treatments were applied according to the following schemes: (1) 30 ppm SAEW for 10 min; or (2) Thymol at concentrations of 32, 64, 128, 256, or 512 μg/mL for 10 min. While for the combination groups, biofilms were first treated with thymol at 32, 64, 128, 256, or 512 μg/mL for 10 min, followed by three washes. Subsequently, 30 ppm SAEW was applied for an additional 10 min. After treatment, ultrasound was used to release bacteria from the biofilm, and the resulting bacterial suspension was serially diluted (10^−1^ to 10^−7^) with 1 × PBS. The diluted suspensions were then plated onto LB agar plates. After incubation, bacterial colonies were counted to evaluate treatment efficacy.

### 2.5 Scanning electron microscopy

To observe bacterial morphology, silicon chips (3 × 3 mm) were placed in a 24-well plate to provide a flat surface for bacterial attachment. Mature biofilms were cultivated on the silicon chips following the previously described experimental procedure. The biofilms were then treated with either SAEW, thymol, or a combination of both, according to the established treatment protocols. After incubation, the silicon chips were rinsed three times with PBS, fixed in 2.5% glutaraldehyde at low temperatures for 15 min, and dehydrated through a series of ethanol concentrations (30%, 50%, 70%, 80%, 90%, and 100%), with each concentration lasting 10 min. Following air drying, the samples were gold-coated and examined using scanning electron microscopy (SEM; Hitachi SU8010, Japan; Liu et al., 2023).

### 2.6 Reactive oxygen species detection

To assess the effect of SAEW on bacterial reactive oxygen species (ROS) levels, a commercial ROS detection kit (Biyuntian Biotechnology Co., Ltd, Shanghai, China) was used. After overnight culture, the bacterial cells were washed three times with PBS and then diluted to an OD_600_ of 0.3–0.4. The bacterial suspension was incubated with the fluorescent probe 2′,7′-dichlorodihydrofluoresce at 37°C in the dark for 30 min (Liu et al., 2023). The monotherapy groups were treated according to the following scheme: (1) 30 ppm SAEW for 10 min; or (2) Thymol at concentrations of 32, 64, 128, 256, or 512 μg/mL for 10 min. The combination groups were treated as follows: first, the biofilms were exposed to thymol at 32, 64, 128, 256, or 512 μg/mL for 10 min, and then treated with 30 ppm SAEW for 10 min. We centrifuge the bacterial cells between different drug (thymol and SAEW) treatments for the next drug treatment. The fluorescence intensity was then measured using a microplate reader (BioTek, Synergy), set to an excitation wavelength of 488 nm and an emission wavelength of 525 nm. The drug concentration was adjusted to 0.5 × Fractional Inhibitory Concentration Index (FICI) values.

### 2.7 RT-qPCR

The relative expression levels of genes associated with *P. aeruginosa* virulence and quorum sensing (including *lasA, lasB, rhlA, pqsA, pqsE*) were analyzed using RT-qPCR (Li et al., 2018). The procedure was performed as previously described (Sonbol et al., 2019). Residual cells from the biofilm were collected and cultured in LB medium at 37°C with shaking overnight to obtain sufficient biomass for RNA extraction. In this study, *rpoB* served as the housekeeping gene, and the 2^−Δ*ΔCt*^ method was used to calculate the expression levels of the virulence genes. RNA extraction was performed following the manufacturer's protocol, and cDNA was synthesized using the Bacterial RNA Miniprep Kit and RevertAid First Strand cDNA Synthesis Kit. PCR amplification was carried out using the TB Green Premix Ex Taq II (Tli RNaseH Plus) kit. The primer sequences used in this study were listed in Supplementary Table S1.

The RT-qPCR running conditions were as follows: (1) Holding stage: 95°C for 30 s; (2) PCR stage: 95°C for 5 s, followed by 50°C for 30 s, for a total of 40 cycles; (3) Melt curve stage: 95°C for 15 s, 60°C for 1 min, and finally 95°C for 15 s.

### 2.8 Surface disinfection of medical devices

We simulated contaminated medical devices using artificially contaminated medical catheters to evaluate whether thymol could enhance the efficacy of SAEW in removing mature biofilms formed on medical instruments (Burton et al., 2006). Briefly, a bacterial suspension with a concentration of 10^6^ CFU/mL was prepared as the initial contaminating bacterial load. A 1 cm-long medical catheter was placed into a 24-well plate. One milliliter of the bacterial suspension was then added to each well, followed by incubation at 37°C for 24 h to allow the formation of mature biofilms on the catheter surfaces. The catheters with attached biofilms were then transferred to a clean 24-well plate and washed to remove planktonic bacteria. After treating with thymol for 10 min, they were washed three times and then treated with SAEW for another 10 min. Na_2_S_2_O3 (0.5%) was added to remove excess chlorine. Finally, 0.9% saline was added to each well, and ultrasonic treatment was applied for 15 min to release bacteria from the biofilms. The resulting bacterial suspension was then serially diluted and inoculated onto LB agar plates for colony counting.

### 2.9 Acute toxicity assessment on mouse skin

Animal research was conducted following ethical standards and approved by the Ethics Committee of the First Affiliated Hospital of Wenzhou Medical University [Approval Number: SYXK(zhe)2021-0017], following the Wenzhou Experimental Animal Welfare and Ethical Standards. A total of 24 ICR mice (comprising three male mice and three female mice per group, with four groups in total) were used in the experiment, employing a single-dose method. The treatment area covered approximately 10% of the body surface for each mouse, specifically a 3 cm × 3 cm depilated area. The monotherapy groups were treated as follows: (1) 30 ppm SAEW was evenly applied to the skin. (2) 512 μg/mL thymol was evenly applied to the skin. In the combination group, 512 μg/mL thymol was applied to the skin for 10 min, followed by the application of 30 ppm SAEW. The skin conditions of the mice were observed at 1, 3, 8, and 24 h after treatment to evaluate for any signs of damage, irritation, or allergic reactions (Na et al., 2020).

### 2.10 Eye irritancy testing on mouse

A total of 24 mice (three male and three female mice per group, with four groups in total) were used for the experiment. During the experiment, the lower eyelid of one eye of each mouse was gently pulled down, and 10 μL of the test substance was instilled into conjunctival sac. The upper and lower eyelids were allowed to passively close for 1 s to prevent sample loss, while another eye served as the control. The monotherapy groups received treatment as follows: (1) 10 μL of 30 ppm SAEW was instilled. (2) 10 μL of 512 μg/mL thymol was instilled. In the combination group, 10 μL of 512 μg/mL thymol was first instilled, followed by 10 μL of 30 ppm SAEW after 10 min. The eyes were not rinsed for 24 h post-instillation. Observations of the eyes were conducted at 1, 24, and 48 h post-instillation to assess for signs of redness, inflammation, cloudiness, or tearing (Zhao et al., 2021).

### 2.11 Statistical analysis

Statistical analysis and graphical representation in this study were performed using Prism 9.0 software (GraphPad Software, LLC; San Diego, California, USA). Data were presented as mean ± standard deviation, based on at least three replicates from three independent experiments. Statistical analyses were performed using Student's *t*-test or ANOVA, with a significance threshold set at *P* < 0.05. The correlation between *P*-values and asterisks is defined as follows: ^*^P < 0.05, ^**^P < 0.01, ^***^P < 0.001, ^****^P < 0.0001.

## 3 Results

### 3.1 The combination of SAEW and thymol significantly eliminates mature biofilms of PAO1

**Figure 1:**
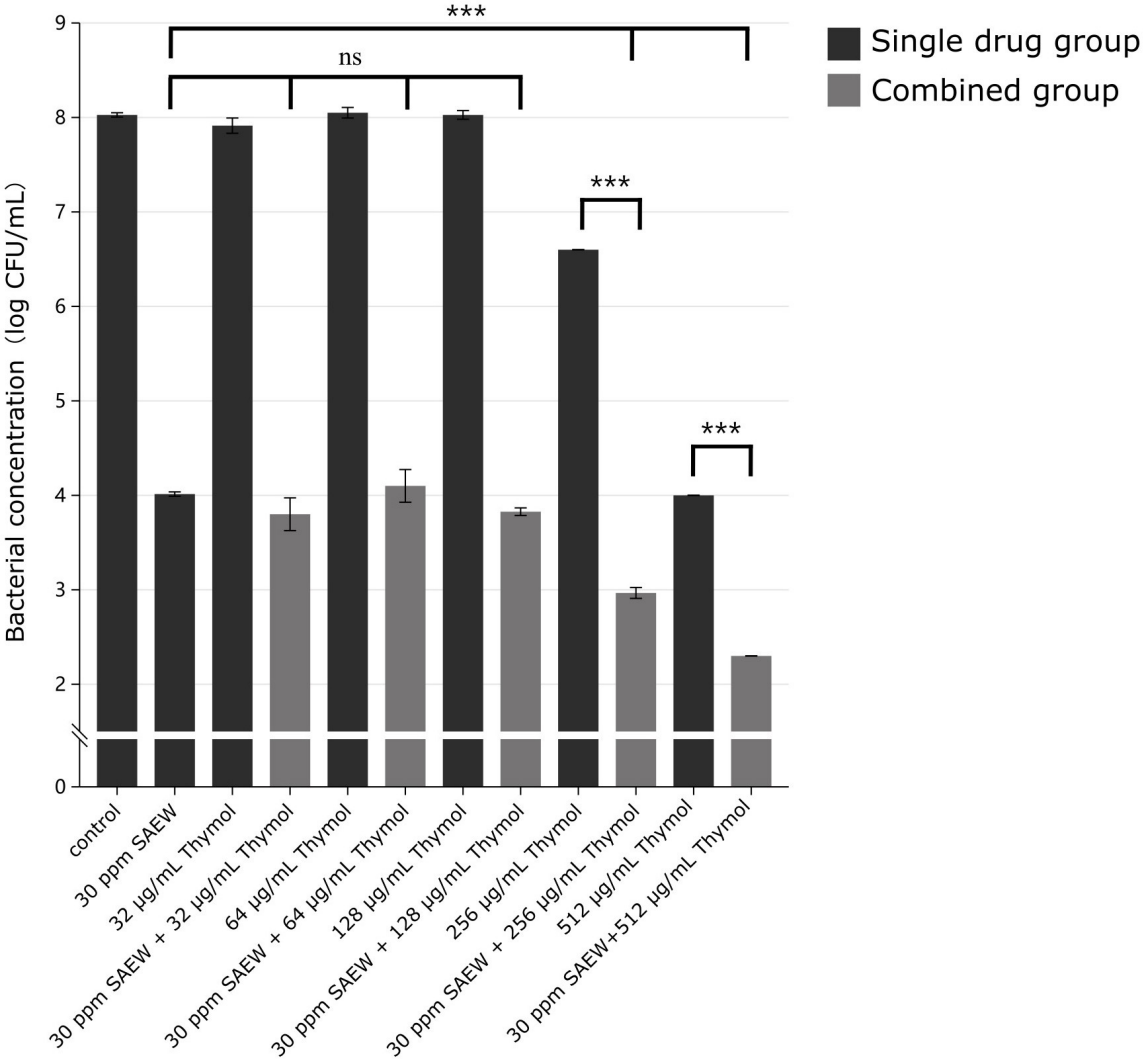
Thymol significantly enhances the ability of SAEW to combat mature biofilms of *P. aeruginosa*. The black bars represent the viable bacterial count in mature biofilms treated with different concentrations of monotherapy; the gray bars represent the viable bacterial count in mature biofilms under different concentrations of combination therapy. The detection limit for bacterial count in this experiment is 10^2^ CFU/mL. ***p < 0.001 were analyzed by multiple comparative *t*-tests.

The combined effect of thymol and SAEW on the elimination of mature PAO1 biofilms was evaluated across various concentrations. The MIC of thymol against *P. aeruginosa* PAO1 was determined to be 256 μg/mL. At sub-inhibitory thymol concentrations ranging from 32 to 128 μg/mL (1/8MIC to 1/2MIC), no enhanced antibiofilm effect was observed when combined with 30 ppm SAEW. However, when the thymol concentration was increased to 256 μg/mL and paired with SAEW, the PAO1 biofilm content significantly decreased by 5.03 log CFU/mL compared to the blank control group. This result demonstrated a substantial enhancement in efficacy, with statistically significant improvements compared to both SAEW alone and the 256 μg/mL thymol treatment groups. Furthermore, at a higher concentration of 512 μg/mL thymol combined with SAEW, the reduction in PAO1 biofilm content reached 5.73 log CFU/mL compared to the blank group, approaching the detection limit of 10^2^ CFU/mL. Consistent with the previous results, this combination demonstrated significant improvement compared to SAEW alone and the 512 μg/mL thymol treatment groups. These results confirm the effectiveness of the thymol-SAEW combination in eliminating PAO1 from mature biofilms (Figure 1).

### 3.2 Scanning electron microscopy reveals that thymol enhanced the antibiofilm effect of SAEW

At 4000 × magnification, scanning electron microscopy clearly demonstrated that the combination of 30 ppm SAEW and 512 μg/mL (2MIC) thymol effectively disrupted the structure of mature biofilms, leading to a significant reduction in bacterial density. This structural alteration indicates the loss of biofilm integrity and potential bacterial viability impairment.

**Figure 2:**
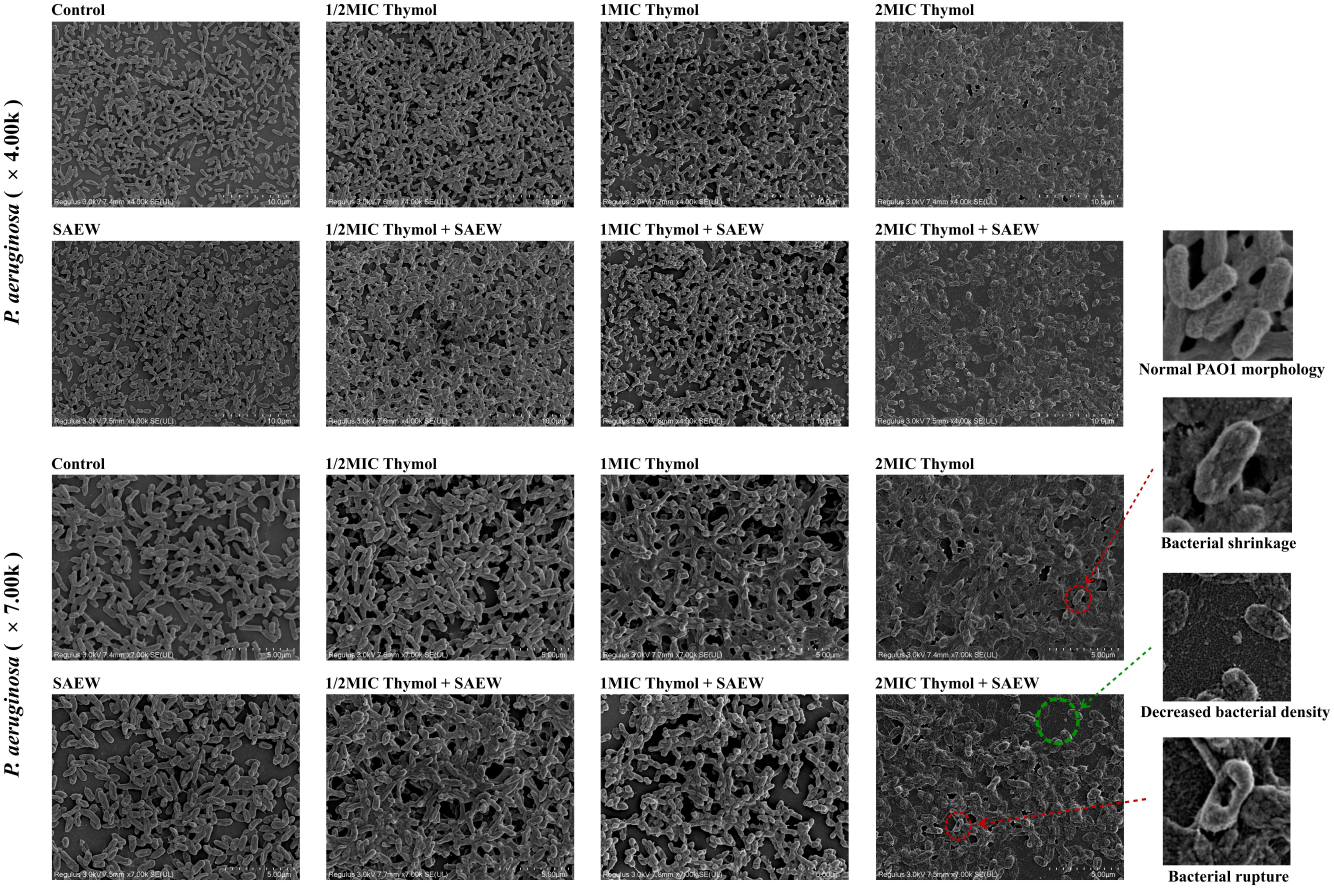
SEM images of bacterial biofilms treated individually or in combination. Red arrows indicate that *P. aeruginosa* shrinks and ruptures under combined treatment; green arrows show that adhesion decreases, bacterial load is reduced, and the biofilm is disrupted under combined treatment.

Further examination at 7000 × magnification provided deeper insights, revealing more pronounced damage to bacterial cells. We observed that *P. aeruginosa* exhibited varying degrees of shrinkage when treated individually with 30 ppm SAEW or different concentrations of thymol; however, no significant bacterial membrane disruption or leakage of intracellular contents was detected. In contrast, when 30 ppm SAEW was combined with 512 μg/mL (2MIC) thymol, the majority of *P. aeruginosa* cells lost their normal structural integrity, displaying severe surface shrinkage and damage, with some cells exhibiting noticeable rupture. These findings suggest that the combined treatment disrupted biofilm cohesion while causing substantial damage at the cellular level. The observations highlight the potent antibiofilm activity of the SAEW-thymol combination, underscoring its potential clinical application in eradicating persistent biofilm-associated infections (Figure 2).

### 3.3 Disinfection of simulated medical catheters

**Figure 3:**
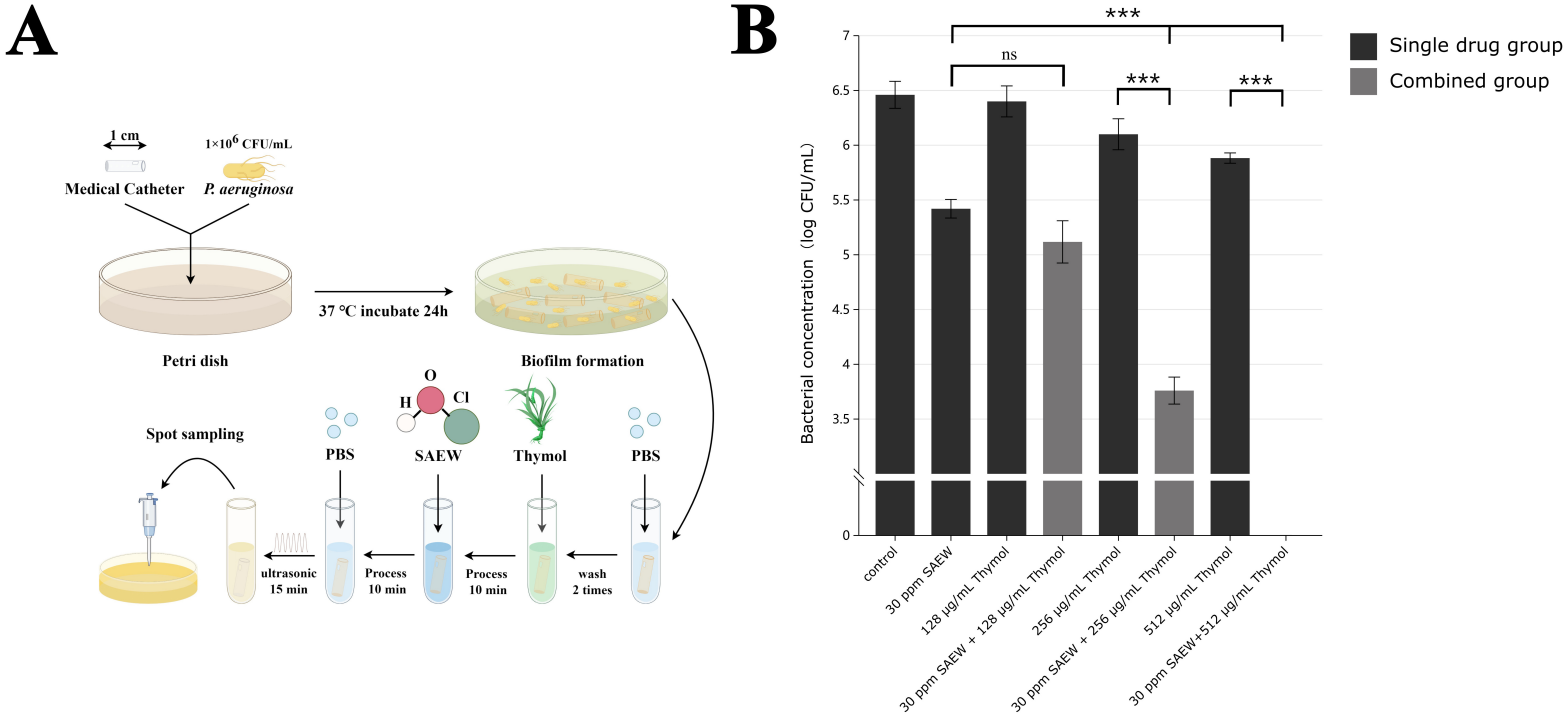
The combination of SAEW and thymol completely eradicates mature biofilms on medical catheters. **(A)** Flowchart of the experiment for the removal of PAO1 mature biofilms on catheters using the combination of SAEW and thymol; **(B)** Experiment for the removal of PAO1 mature biofilms on catheters using SAEW combined with thymol. The black bars represent the viable bacterial counts in mature biofilms treated with different concentrations of monotherapy, while the gray bars indicate the viable bacterial counts in mature biofilms treated with different concentrations of the combination. The detection limit for bacterial counts in this experiment is 10^2^ CFU/mL. ***p < 0.001 were analyzed by multiple comparative *t*-tests.

The experimental procedure, as illustrated in Figure 3A, involved biofilm formation on medical catheters and subsequent treatments with SAEW and thymol to assess their antibiofilm efficacy. The results indicated that the combination of 128 μg/mL thymol (1/2 × MIC) with 30 ppm SAEW did not enhance the antibiofilm effect. However, at higher concentrations of 256 μg/mL (1 × MIC) and 512 μg/mL (2 × MIC), the combination with SAEW demonstrated significant efficacy in reducing the biofilms on the catheters. Specifically, the combination of 256 μg/mL thymol and SAEW reduced the bacterial load in the catheter biofilms by 2.7 log CFU/mL. Remarkably, the combination of 512 μg/mL thymol with SAEW achieved complete eradication of the biofilms within the catheters, underscoring its potential as a powerful strategy for managing biofilm-associated infections (Figure 3B).

### 3.4 ROS quantification

**Figure 4:**
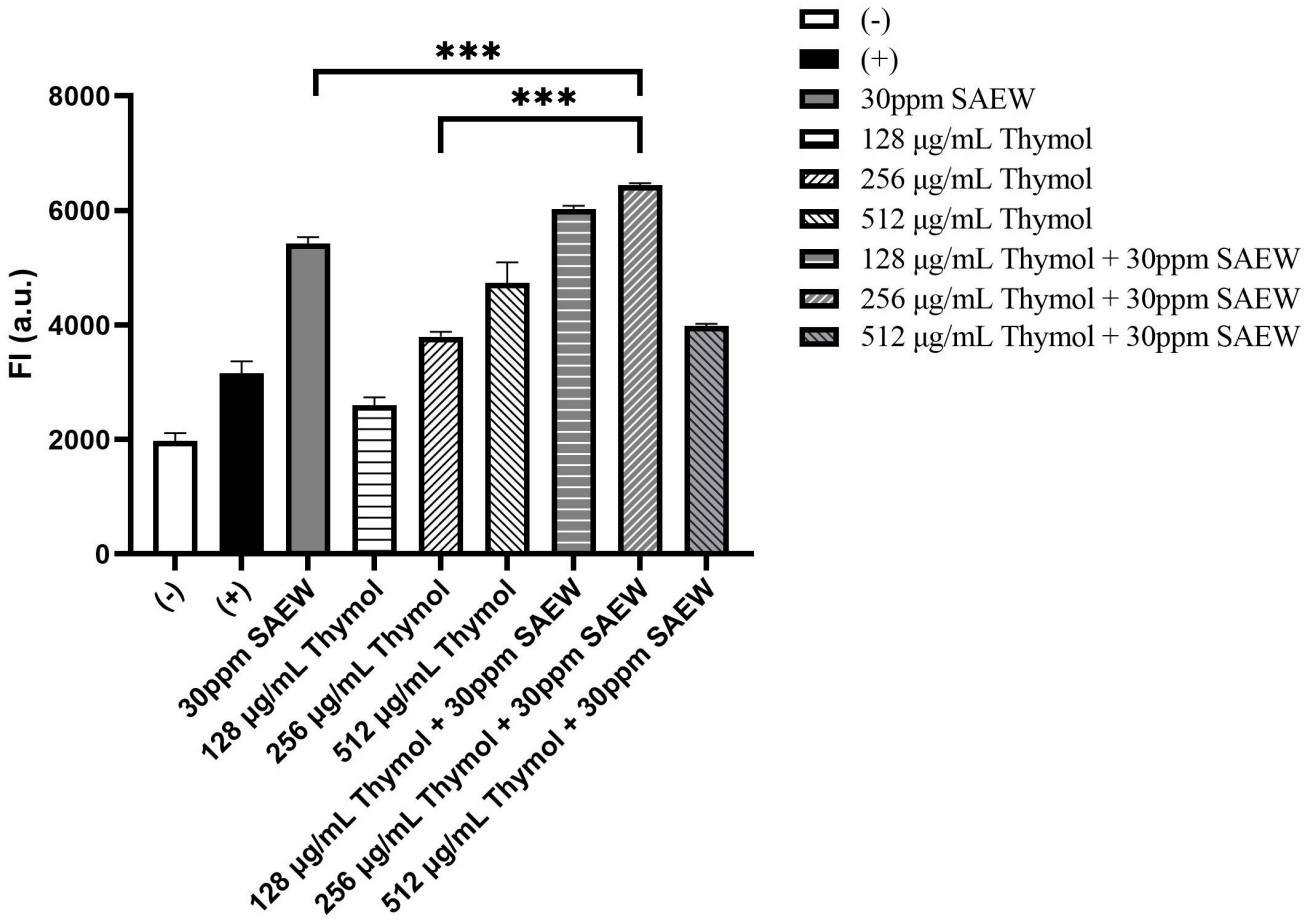
Changes of ROS levels during the removal of mature biofilms using SAEW combined with thymol. (−) represents the negative control, which is the untreated blank group; (+) represents the positive control, which is the group treated with the positive control reagent included in the ROS assay kit. ***p < 0.001 were analyzed by multiple comparative *t*-tests.

The positive control from the ROS assay kit validated the experimental procedures, as treatment with this reagent resulted in a significant increase in ROS levels in PAO1 compared to the negative control group. Treatment with SAEW alone also resulted in a notable increase in ROS levels. Similarly, thymol treatments at concentrations of 128, 256, and 512 μg/mL demonstrated a dose-dependent elevation in ROS levels. When 128 or 256 μg/mL of thymol were combined with SAEW, ROS levels were significantly higher compared to their respective monotherapy groups. However, for the combination of 512 μg/mL thymol with SAEW, the ROS levels were lower than those observed in the monotherapy groups. This reduction is likely due to the effective bactericidal action of the combination treatment, which caused cell lysis and the subsequent loss of intracellular fluorescent probes (Figure 4).

### 3.5 RT-qPCR detection of virulence gene expression in *P. aeruginosa*

**Figure 5:**
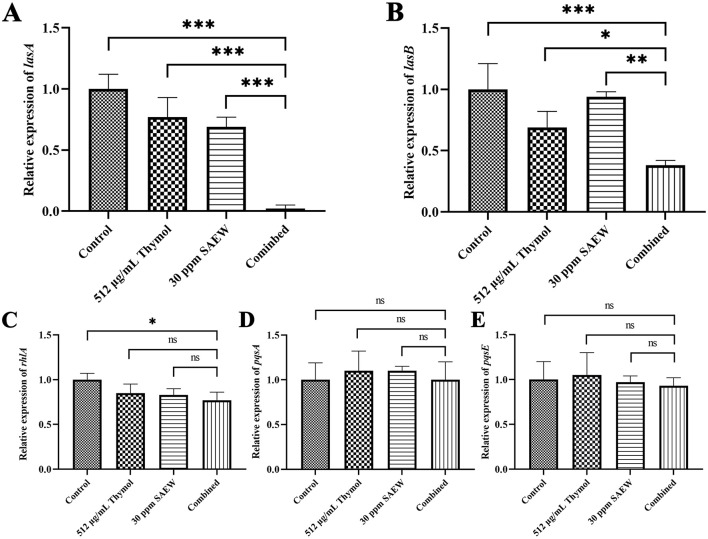
The combination of SAEW and thymol can reduce the expression of virulence genes (*lasA* and *lasB*) in *P. aeruginosa*. **(A–E)** Relative expression levels of *lasA, lasB, rhlA, pqsA*, and *pqsE* in *P. aeruginosa* under treatment with SAEW and thymol, either alone or in combination. The relative expression analysis of each gene standardizes the gene expression level of the untreated group to “1”, and calculates the gene expression fold of the treated group compared to the untreated group. *p < 0.05, **p < 0.01, ***p < 0.001 were analyzed by multiple comparative *t*-tests.

Previous studies have shown that *P. aeruginosa* has a remarkable ability to degrade elastin, which is a crucial component of connective tissue. This capability likely underpins the bacterial pathogenicity and its ability to persist within human tissues. The extracellular enzyme LasA plays a pivotal role in this process, as it is essential for the bacterial elastase activity. Furthermore, the secreted enzyme LasB is recognized as a significant virulence factor that not only contributes to tissue degradation but also enhances the processing of LasA, thereby increasing its elastolytic effectiveness (Toder et al., 1991; Kessler et al., 1993; Camberlein et al., 2022; Llanos et al., 2023). Additionally, LasA and LasB are closely associated with the quorum sensing of *P. aeruginosa* and are commonly used to evaluate the quorum sensing inhibitory effects of drugs (Li et al., 2018). In our study, the combined treatment group exhibited a noteworthy decrease in the relative expression levels of the virulence genes *lasA* and *lasB* when compared to both the control and monotherapy groups. This reduction highlights the efficacy of the combined treatment in mitigating key virulence factors that facilitate tissue invasion and damage. Conversely, the treatment did not significantly affect the expression levels of other related genes, such as *rhlA, pqsA*, or *pqsE*. This specificity suggests that the combined approach effectively targets specific pathways involved in virulence without broadly impacting other regulatory mechanisms related to the pathogenic potential of *P. aeruginosa*. These results underscore the therapeutic potential of targeting LasA and LasB as a focused strategy for combating infections caused by this opportunistic pathogen (Figure 5).

### 3.6 Safety evaluation of SAEW combined with thymol

**Figure 6:**
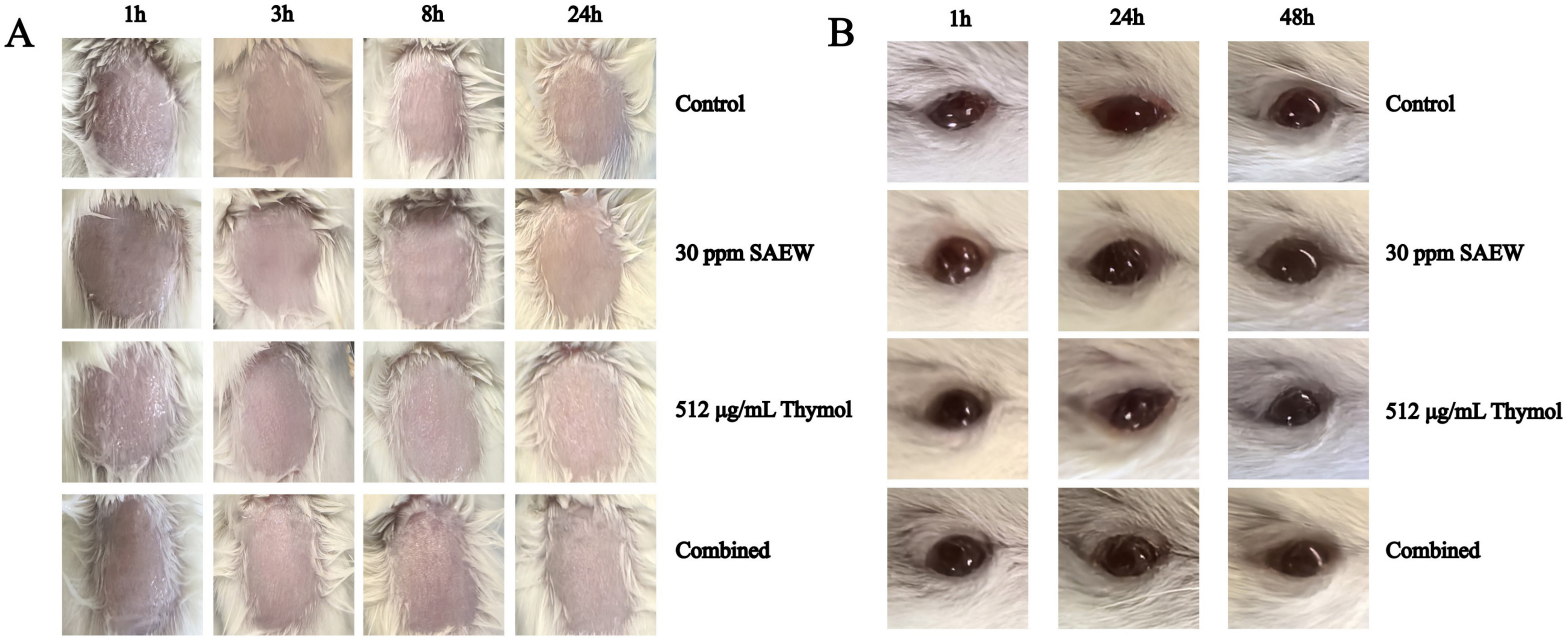
The combination of SAEW and thymol demonstrates good safety. **(A)** Acute skin toxicity experiment on mice; **(B)** Mouse eye irritation experiment.

The acute dermal toxicity test in mice showed no signs of redness, lesions, or erythema on the skin of mice treated with SAEW, thymol, or their combination during the 1–24-h observation period, compared to the control group. This indicates that the combined application of SAEW and thymol does not cause skin irritation (Figure 6A). Similarly, the ocular irritation test revealed no signs of redness, inflammation, cloudiness, or tearing in the eyes of mice treated with either SAEW, thymol, or their combination or over the 1–48-h observation period, compared to the control group. These findings confirm that the combined use of SAEW and thymol is safe and does not induce ocular irritation. Overall, the results highlight the potential of this combination as a safe disinfectant for clinical applications (Figure 6B).

## 4 Discussion

The clinical threat posed by *P. aeruginosa* is increasingly severe, particularly evident in healthcare-associated infections (Kerr and Snelling, 2009). The formation of biofilms is a critical factor contributing to its pathogenicity, allowing the bacteria to firmly adhere to medical devices and human tissues, while resisting host immune responses and antimicrobial treatments (Costerton et al., 1999; Thi et al., 2020). This biofilm structure not only enables persistent and recurrent infections but also facilitates the acquisition of antimicrobial resistance through gene transfer, further complicating treatment (Zheng et al., 2023). In scenarios such as ventilator-associated pneumonia and catheter-associated urinary tract infections, the biofilm of *P. aeruginosa* plays a significant role, markedly increasing the severity of infections (Maurice et al., 2018; Govindan Nadar et al., 2021). Therefore, effective measures for the removal of these biofilms are urgently needed to reduce the risk of transmission in hospital environments and improve clinical outcomes for patients. In this context, the development of novel disinfectants, particularly those designed to target biofilms, will be crucial in addressing this challenge. Currently, other antibiofilm therapies, although effective in biofilm eradication, often require extended treatment durations, such as the application of plant extracts as biofilm disruptors (Alam et al., 2020). However, the combination therapy of SAEW and thymol demonstrates a potent biofilm-clearing effect within a short period, enhancing the efficiency of biofilm removal while addressing the practical requirements for environmental applications.

SAEW represents a promising advancement in disinfection technology, offering distinct characteristics and advantages over traditional disinfectants (Ye et al., 2017). Its primary active component, hypochlorous acid, enables rapid eradication of a wide spectrum of pathogens, including bacteria, fungi, and viruses, without leaving harmful residues. Unlike conventional disinfectants such as sodium hypochlorite and ethanol, SAEW does not corrode surfaces or irritate the eyes, skin, or respiratory tract, making it ideal for various applications in food, healthcare, and environmental cleaning (Hao et al., 2013; Zang et al., 2019; Du et al., 2024). However, despite its broad-spectrum efficacy and minimal environmental impact, SAEW is not without limitations. Similar to other chlorine-based disinfectants, its dependence on oxidative mechanisms raises concerns about the potential emergence of disinfectant-resistant bacteria, which could also develop cross-resistance to clinical antimicrobial agents (Bland et al., 2021). Moreover, the emergence of strains resistant to chlorine-based agents poses a significant challenge to the effectiveness of SAEW, highlighting the need for a critical evaluation of its application in clinical settings (Russell, 1986). To address these challenges and enhance the overall effectiveness of SAEW, combining it with non-antimicrobial agents could be a pivotal strategy. This combination application method could improve antimicrobial efficacy while reducing the risk of resistance development, offering a safer and more effective solution for infection control in healthcare and other applications. Currently, there are studies that combine SAEW with other substances, such as using Didecyldimethylammonium bromide in conjunction with SAEW to combat *Staphylococcus aureus* and *P. aeruginosa* biofilms (Li et al., 2022). However, there is limited research on the combined application of SAEW and QSI.

Building upon the aforementioned information, we report for the first time that thymol enhances the antimicrobial and antibiofilm effects of SAEW, and we elucidate the underlying mechanisms. In our preliminary experiments, we found that mixing SAEW with thymol reduced the available chlorine concentration in SAEW, thereby affecting its efficacy. Moreover, numerous studies have reported the combined use of thymol with acidic substances, suggesting that a low-pH treatment environment may have limited impact on thymol activity (Chung et al., 2023; Li et al., 2025). Nevertheless, it remains important to minimize any potential influence of pH on thymol's effectiveness. To avoid the drawbacks of combining the two, we adopted a sequential application approach. The order of SAEW and thymol treatment was determined based on practical clinical applications. Given that the available chlorine in SAEW has sustained antimicrobial activity, and to prevent possible pH-induced interference with thymol's activity, SAEW was applied after thymol treatment in practice to preserve its prolonged antibacterial effect. Therefore, we implemented an experimental procedure in which thymol treatment was followed by SAEW treatment. The combination of thymol and SAEW has demonstrated remarkable effectiveness in combating *P. aeruginosa*, particularly in the context of mature biofilm eradication. At optimal concentrations, this combination application method significantly reduced the biomass of PAO1 biofilms, showcasing its ability to penetrate and disrupt established biofilm structures. Furthermore, the combination treatment effectively eradicated mature biofilms from medical catheters, highlighting its practical potential in clinical settings where biofilm-associated infections pose significant challenges. Additionally, we utilized SEM to provide a more intuitive visualization of the bactericidal and biofilm-clearing effects of SAEW and thymol. Our observations revealed a substantial reduction in *P. aeruginosa* density following combined treatment, along with drastic morphological changes, including severe cell shrinkage and membrane disruption, with some bacteria exhibiting evident rupture.

In terms of the combined antimicrobial and antibiofilm mechanisms of SAEW and thymol, the intrinsic antimicrobial potential of thymol itself should not be overlooked. Previous studies have demonstrated that thymol can cause bacterial cell membrane destabilization, leakage of cytoplasmic contents, and DNA damage, which has led to numerous investigations on the synergistic antimicrobial effects of thymol combined with other agents (Marchese et al., 2016; Chung et al., 2023; Peter et al., 2024). In this study, we focused on explaining the antimicrobial mechanism of the SAEW-thymol combination through changes in intracellular reactive oxygen species (ROS) levels in bacteria. Numerous studies have demonstrated that SAEW can mediate its bactericidal effects by disrupting bacterial ROS homeostasis through the inhibition of intracellular antioxidant enzyme activity (Ye et al., 2017; Li H. et al., 2021; Wu et al., 2022). Consistent with previous findings, our ROS assay results showed that SAEW alone significantly increased intracellular ROS levels in *P. aeruginosa*. However, we found that in the presence of thymol, the combination of SAEW and thymol led to a significantly greater accumulation of intracellular ROS compared to SAEW treatment alone. This indicates that enhanced antimicrobial activity of the SAEW-thymol combination is largely attributable to the enhanced accumulation of intracellular ROS, thereby disturbing ROS homeostasis. Notably, the ROS fluorescence intensity in the 512 μg/mL thymol combination group was lower than that in the 256 μg/mL thymol combination group. Based on TEM images, we observed that under treatment with 512 μg/mL thymol combination group, a large number of bacterial cells exhibited pronounced shrinkage or even rupture, which may have led to the leakage of fluorescent substances from the cells, thereby reducing the detected ROS fluorescence intensity.

The combination of thymol and SAEW exhibited strong antibacterial activity against *P. aeruginosa* while significantly affecting the expression of virulence genes associated with this pathogen. Specifically, studies have shown that the *lasA* and *lasB* genes are crucial for the elastase activity of *P. aeruginosa*, which enables the degradation of elastin, a key component of connective tissue. Consequently, *lasA* and *lasB* are closely linked to the tissue-invasive virulence of *P. aeruginosa* (Toder et al., 1991; Kessler et al., 1993). Notably, as members of the *las* system, *lasA* and *lasB* also play an essential role in *P. aeruginosa* QS system (Li et al., 2018). In addition, studies have demonstrated that thymol, as a QSI, not only suppresses the QS system and biofilm formation of various bacteria, but also downregulates the expression of adhesion-related genes in *E. coli* and *Salmonella Enteritidis* (Upadhyaya et al., 2013; Singh et al., 2017; Saptami et al., 2022; Goodarzi et al., 2023). Our results indicate that thymol can independently suppress the expression of *lasA* and *lasB*. Moreover, SAEW also affects the expression of these genes. However, the combined application of SAEW and thymol results in a further reduction in their expression levels compared to either agent alone, maintaining consistently low levels. Thus, through the joint suppression of *lasA* and *lasB*, the combination of thymol and SAEW not only inhibits the QS system of *P. aeruginosa* but also reduces tissue-invasive virulence. In addition, *pqsA* and *pqsR* play important roles in the pseudomonas quinolone signal (PQS) system and are involved in the QS regulation of *P. aeruginosa* (Sabir et al., 2020). Similarly, *rhlA* is a virulence-associated gene that is also closely linked to the QS system of *P. aeruginosa* (Wang et al., 2020). However, we found that the combination of thymol and SAEW did not significantly inhibit the expression of *rhlA, pqsA*, and *pqsE*. This may be attributed to the fact that neither thymol nor SAEW individually affects the expression of these genes, suggesting that the enhanced anti-virulence effect of the combination mainly relies on their intrinsic activities. It is noteworthy that while investigating the expression of PAO1 virulence genes under the combined treatment, we resuspended residual biofilm bacteria in LB medium and assessed their virulence after regrowth. Surprisingly, these sub-damaged bacteria still exhibited reduced expression of QS-associated virulence genes (*lasA* and *lasB*) upon regrowth. This finding suggests that combined application of SAEW and thymol has a strong and lasting impact on virulence suppression. Therefore, this dual-targeting approach simultaneously inhibiting microbial QS and virulence may lead to more effective infection management strategies, particularly in hospital settings where *P. aeruginosa* poses a significant threat.

Importantly, this combination therapy has demonstrated a favorable safety profile, with no significant adverse effects observed during acute toxicity assessments. This characteristic makes the thymol and SAEW combination an attractive candidate for clinical use, providing a dual benefit of effective disinfection while maintaining patient safety. Overall, the combined application of thymol and SAEW presents a promising strategy for managing *P. aeruginosa* infections, particularly in environments where biofilm formation poses a significant threat. However, this study has some limitations. Our preliminary research indicated that due to the unstable and easily decomposable chemical properties of SAEW, mixing SAEW with thymol would reduce the available chlorine concentration, thereby diminishing its bactericidal effect. Therefore, we adopted a stepwise application strategy in this study. Currently, many studies on the combined application of SAEW also utilize a stepwise approach (Li et al., 2022). Therefore, we hope that future research can address the instability of SAEW through emerging technologies, such as using nanocapsule carriers, and develop an efficient environmental composite disinfectant that can be mixed with SAEW (Wen et al., 2023; Wang et al., 2025). This will significantly improve disinfection effectiveness and convenience, making it more suitable for clinical applications.

## 5 Conclusions

This study demonstrated the efficacy of thymol combined with SAEW in eliminating mature biofilms of *P. aeruginosa* PAO1, which was further validated in medical catheters. SEM analysis revealed that the combined treatment caused significant bacterial shrinkage and rupture. Mechanistically, the combination facilitates bacterial cell death by further promoting SAEW-mediated intracellular ROS accumulation. In terms of virulence reduction, the combination did not affect the expression of *pqsA, pqsE*, or *rhlA* in *P. aeruginosa*. However, it significantly suppressed the expression of key virulence and QS-related genes *lasA* and *lasB*, which may contribute to the inhibition of the QS system and tissue-invasive virulence of *P. aeruginosa*. Regarding safety, no signs of skin or ocular toxicity were observed in mice under this treatment. These findings suggest that the combination of SAEW and thymol could serve as a safe and effective strategy for biofilm eradication and infection control.

## Data Availability

The raw data supporting the conclusions of this article will be made available by the authors, without undue reservation.

## References

[B1] Alam K. Farraj D. A. A. Mah E. F. S. Yameen M. A. Elshikh M. S. Alkufeidy R. M.. (2020). Anti-biofilm activity of plant derived extracts against infectious pathogen-*Pseudomonas aeruginosa* PAO1. J. Infect. Public Health 13, 1734–1741. 10.1016/j.jiph.2020.07.007 32753311

[B2] Bland R. Waite-Cusic J. Weisberg A. J. Riutta E. R. Chang J. H. Kovacevic J.. (2021). Adaptation to a commercial quaternary ammonium compound sanitizer leads to cross-resistance to select antibiotics in *Listeria monocytogenes* isolated from fresh produce environments. Front. Microbiol. 12:782920. 10.3389/fmicb.2021.782920 35082767 PMC8784610

[B3] Burton E. Gawande P. V. Yakandawala N. Lovetri K. Zhanel G. G. Romeo T.. (2006). Antibiofilm activity of GlmU enzyme inhibitors against catheter-associated uropathogens. Antimicrob. Agents Chemother. 50, 1835–1840. 10.1128/AAC.50.5.1835-1840.2006 16641457 PMC1472218

[B4] Camberlein V. Jézéquel G. Haupenthal J. Hirsch A. K. H. (2022). The structures and binding modes of small-molecule inhibitors of *Pseudomonas aeruginosa* Elastase LasB. Antibiotics 11:1060. 10.3390/antibiotics11081060 36009930 PMC9404851

[B5] Cao W. Zhu Z. W. Shi Z. X. Wang C. Y. Li B. M. (2009). Efficiency of slightly acidic electrolyzed water for inactivation of *Salmonella enteritidis* and its contaminated shell eggs. Int. J. Food Microbiol. 130, 88–93. 10.1016/j.ijfoodmicro.2008.12.021 19185376

[B6] Chung S. Y. Cho T. J. Yu H. Park S. G. Kim S. R. Kim S. A.. (2023). Efficacy of combined caprylic acid and thymol treatments for inactivation of *Listeria monocytogenes* on enoki mushrooms in household and food-service establishments. Food Res. Int. 166, 112601. 10.1016/j.foodres.2023.112601 36914348

[B7] Costerton J. W. Stewart P. S. Greenberg E. P. (1999). Bacterial biofilms: a common cause of persistent infections. Science 284, 1318–1322. 10.1126/science.284.5418.1318 10334980

[B8] Deryabin D. Galadzhieva A. Kosyan D. Duskaev G. (2019). Plant-derived inhibitors of AHL-mediated quorum sensing in bacteria: modes of action. Int. J. Mol. Sci. 20:5588. 10.3390/ijms20225588 31717364 PMC6888686

[B9] Du Y. Tian Q. Li G. Yi J. Hu X. Jiang Y.. (2024). Advanced application of slightly acidic electrolyzed water for fresh-cut fruits and vegetables preservation. Food Res. Int. 195:114996. 10.1016/j.foodres.2024.114996 39277256

[B10] Goodarzi R. Yousefimashouf R. Sedighi I. Moradi A. Taheri M. (2023). Effect of thymol on antimicrobial susceptibility, and adhesion genes expression of uropathogenic *Escherichia coli* isolated from pediatric urinary tract infection. J. Pediatr. Urol. 19, 654.e651–654.e657. 10.1016/j.jpurol.2023.07.001 37481428

[B11] Govindan Nadar R. Chackaravarthy G. Ramachandran G. Manoharan N. Muhammad Zubair S. Alharbi N. S.. (2021). Isolation and molecular identification of biofilm producing *P. aeruginosa* and *K. pneumoniae* from urinary tract infections patient urine sample. J. Infect. Public Health 14, 1875–1880. 10.1016/j.jiph.2021.11.004 34802975

[B12] Hao X. X. Li B. M. Zhang Q. Lin B. Ge L. P. Wang C. Y.. (2013). Disinfection effectiveness of slightly acidic electrolysed water in swine barns. J. Appl. Microbiol. 115, 703–710. 10.1111/jam.12274 23742207

[B13] Humphries R. Bobenchik A. M. Hindler J. A. Schuetz A. N. (2021). Overview of changes to the clinical and laboratory standards institute performance standards for antimicrobial susceptibility testing, M100, 31st edition. J. Clin. Microbiol. 59:e0021321. 10.1128/JCM.00213-21 34550809 PMC8601225

[B14] Jeong G. J. Khan F. Tabassum N. Kim Y. M. (2024). Natural and synthetic molecules with potential to enhance biofilm formation and virulence properties in *Pseudomonas aeruginosa*. Crit. Rev. Microbiol. 50, 830–858. 10.1080/1040841X.2023.2282459 37968960

[B15] Kerr K. G. Snelling A. M. (2009). *Pseudomonas aeruginosa*: a formidable and ever-present adversary. J. Hosp. Infect. 73, 338–344. 10.1016/j.jhin.2009.04.020 19699552

[B16] Kessler E. Safrin M. Olson J. C. Ohman D. E. (1993). Secreted LasA of *Pseudomonas aeruginosa* is a staphylolytic protease. J. Biol. Chem. 268, 7503–7508. 10.1016/S0021-9258(18)53203-8 8463280

[B17] Kim H. S. Choi S. J. Yoon K. S. (2018). Efficacy evaluation of control measures on the reduction of *Staphylococcus aureus* in Salad and *Bacillus cereus* in fried rice served at restaurants. Foodborne Pathog. Dis. 15, 198–209. 10.1089/fpd.2017.2334 29265878

[B18] Kurahashi M. Ito T. Naka A. (2021). Spatial disinfection potential of slightly acidic electrolyzed water. PLoS ONE 16:e0253595. 10.1371/journal.pone.0253595 34214092 PMC8253431

[B19] Li H. Liang D. Huang J. Cui C. Rao H. Zhao D.. (2021). The bactericidal efficacy and the mechanism of action of slightly acidic electrolyzed water on *Listeria monocytogenes*' survival. Foods 10:2671. 10.3390/foods10112671 34828952 PMC8621911

[B20] Li L. Mu T. H. Zhang M. (2021). Contribution of ultrasound and slightly acid electrolytic water combination on inactivating *Rhizopus stolonifer* in sweet potato. Ultrason. Sonochem. 73:105528. 10.1016/j.ultsonch.2021.105528 33773434 PMC8027897

[B21] Li R. Ding X. Lei M. Li P. Giannenas I. Wang J.. (2025). The impact of combined thymol and rosmarinic acid on the intestinal microbiota and barrier function of the piglets challenged by *Escherichia coli* K88. Anim. Nutr. 20, 131–144. 10.1016/j.aninu.2024.11.008 39967693 PMC11834115

[B22] Li W. R. Ma Y. K. Xie X. B. Shi Q. S. Wen X. Sun T. L.. (2018). Diallyl disulfide from garlic oil inhibits *Pseudomonas aeruginosa* quorum sensing systems and corresponding virulence factors. Front. Microbiol. 9:3222. 10.3389/fmicb.2018.03222 30666240 PMC6330763

[B23] Li Y. Wang H. Zheng X. Li Z. Wang M. Luo K.. (2022). Didecyldimethylammonium bromide: application to control biofilms of *Staphylococcus aureus* and *Pseudomonas aeruginosa* alone and in combination with slightly acidic electrolyzed water. Food Res. Int. 157:111236. 10.1016/j.foodres.2022.111236 35761549

[B24] Liu H. Huang Z. Chen H. Zhang Y. Yu P. Hu P.. (2023). A potential strategy against clinical carbapenem-resistant Enterobacteriaceae: antimicrobial activity study of sweetener-decorated gold nanoparticles *in vitro* and *in vivo*. J. Nanobiotechnol. 21:409. 10.1186/s12951-023-02149-x 37932843 PMC10626710

[B25] Llanos A. Achard P. Bousquet J. Lozano C. Zalacain M. Sable C.. (2023). Higher levels of *Pseudomonas aeruginosa* LasB elastase expression are associated with early-stage infection in cystic fibrosis patients. Sci. Rep. 13:14208. 10.1038/s41598-023-41333-9 37648735 PMC10468528

[B26] Luo K. Oh D. H. (2016). Inactivation kinetics of *Listeria monocytogenes* and Salmonella enterica serovar Typhimurium on fresh-cut bell pepper treated with slightly acidic electrolyzed water combined with ultrasound and mild heat. Food Microbiol. 53, 165–171. 10.1016/j.fm.2015.09.014 26678144

[B27] Marchese A. Orhan I. E. Daglia M. Barbieri R. Di Lorenzo A. Nabavi S. F.. (2016). Antibacterial and antifungal activities of thymol: a brief review of the literature. Food Chem. 210, 402–414. 10.1016/j.foodchem.2016.04.111 27211664

[B28] Maurice N. M. Bedi B. Sadikot R. T. (2018). *Pseudomonas aeruginosa* biofilms: host response and clinical implications in lung infections. Am. J. Respir. Cell Mol. Biol. 58, 428–439. 10.1165/rcmb.2017-0321TR 29372812 PMC5894500

[B29] Na Y. G. Kim M. Han M. Huh H. W. Kim J. S. Kim J. C.. (2020). Characterization of hepatitis b surface antigen loaded polylactic acid-based microneedle and its dermal safety profile. Pharmaceutics 12:531. 10.3390/pharmaceutics12060531 32527003 PMC7355901

[B30] Ni L. Cao W. Zheng W. C. Zhang Q. Li B. M. (2015). Reduction of microbial contamination on the surfaces of layer houses using slightly acidic electrolyzed water. Poult. Sci. 94, 2838–2848. 10.3382/ps/pev261 26371328

[B31] Peter S. Sotondoshe N. Aderibigbe B. A. (2024). Carvacrol and thymol hybrids: potential anticancer and antibacterial therapeutics. Molecules 29:2277. 10.3390/molecules29102277 38792138 PMC11123974

[B32] Russell A. D. (1986). Chlorhexidine: antibacterial action and bacterial resistance. Infection 14, 212–215. 10.1007/BF01644264 3539812

[B33] Sabir S. Subramoni S. Das T. Black D. S. Rice S. A. Kumar N.. (2020). Design, synthesis and biological evaluation of novel anthraniloyl-AMP mimics as PQS biosynthesis inhibitors against *Pseudomonas aeruginosa* resistance. Molecules 25:3103. 10.3390/molecules25133103 32646050 PMC7412332

[B34] Saptami K. Arokia Balaya Rex D. Chandrasekaran J. Rekha P. D. (2022). Competitive interaction of thymol with cviR inhibits quorum sensing and associated biofilm formation in Chromobacterium violaceum. Int. Microbiol. 25, 629–638. 10.1007/s10123-022-00247-8 35554762

[B35] Singh A. Gupta R. Tandon S. Pandey R. (2017). Thyme oil reduces biofilm formation and impairs virulence of *Xanthomonas oryzae*. Front. Microbiol. 8:1074. 10.3389/fmicb.2017.01074 28659894 PMC5468448

[B36] Sonbol F. I. El-Banna T. E. Abd El-Aziz A. A. El-Ekhnawy E. (2019). Impact of triclosan adaptation on membrane properties, efflux and antimicrobial resistance of *Escherichia coli* clinical isolates. J. Appl. Microbiol. 126, 730–739. 10.1111/jam.14158 30431693

[B37] Tango C. N. Mansur A. R. Kim G. H. Oh D. H. (2014). Synergetic effect of combined fumaric acid and slightly acidic electrolysed water on the inactivation of food-borne pathogens and extending the shelf life of fresh beef. J. Appl. Microbiol. 117, 1709–1720. 10.1111/jam.12658 25273314

[B38] Thi M. T. T. Wibowo D. Rehm B. H. A. (2020). *Pseudomonas aeruginosa* biofilms. Int. J. Mol. Sci. 21:8671. 10.3390/ijms21228671 33212950 PMC7698413

[B39] Toder D. S. Gambello M. J. Iglewski B. H. (1991). *Pseudomonas aeruginosa* LasA: a second elastase under the transcriptional control of lasR. Mol. Microbiol. 5, 2003–2010. 10.1111/j.1365-2958.1991.tb00822.x 1766376

[B40] Upadhyaya I. Upadhyay A. Kollanoor-Johny A. Darre M. J. Venkitanarayanan K. (2013). Effect of plant derived antimicrobials on *Salmonella enteritidis* adhesion to and invasion of primary chicken oviduct epithelial cells *in vitro* and virulence gene expression. Int. J. Mol. Sci. 14, 10608–10625. 10.3390/ijms140510608 23698782 PMC3676857

[B41] Walczak M. Michalska-Sionkowska M. Olkiewicz D. Tarnawska P. Warżyńska O. (2021). Potential of carvacrol and thymol in reducing biofilm formation on technical surfaces. Molecules 26:2723. 10.3390/molecules26092723 34066411 PMC8125478

[B42] Wang J. Wu Z. Ma X. Huang Z. Dong H. Zhang J.. (2025). NIR-II emissive biohybrid nanovesicles as mild-temperature photothermal antibiofilm agents against acute bacterial skin and skin-structure infections. Interdiscipl. Med. 3:e20240053. 10.1002/INMD.20240053

[B43] Wang M. Zhao L. Wu H. Zhao C. Gong Q. Yu W.. (2020). Cladodionen is a potential quorum sensing inhibitor against *Pseudomonas aeruginosa*. Mar. Drugs 18:205. 10.3390/md18040205 32290259 PMC7230538

[B44] Wen M. Wang J. Ou Z. Nie G. Chen Y. Li M.. (2023). Bacterial extracellular vesicles: a position paper by the microbial vesicles task force of the Chinese society for extracellular vesicles. Interdiscipl. Med. 1:e20230017. 10.1002/INMD.2023001734035881

[B45] Wu X. Wu C. Lu D. Wu Y. Ye Z. Xia L.. (2022). Variation of soil microbial community and sterilization to *Fusarium oxysporum* f. sp. niveum play roles in slightly acidic electrolyzed water-alleviated watermelon continuous cropping obstacle. Front. Microbiol. 13:837121. 10.3389/fmicb.2022.837121 35572699 PMC9097028

[B46] Yan P. Chelliah R. Jo K. H. Selvakumar V. Chen X. Jo H. Y.. (2022). Stability and antibiofilm efficiency of slightly acidic electrolyzed water against mixed-species of *Listeria monocytogenes* and *Staphylococcus aureus*. Front. Microbiol. 13:865918. 10.3389/fmicb.2022.865918 35633663 PMC9135065

[B47] Yan P. Daliri E. B. Oh D. H. (2021). New clinical applications of electrolyzed water: a review. Microorganisms 9:136. 10.3390/microorganisms9010136 33435548 PMC7827692

[B48] Ye Z. Wang S. Chen T. Gao W. Zhu S. He J.. (2017). Inactivation mechanism of *Escherichia coli* induced by slightly acidic electrolyzed water. Sci. Rep. 7:6279. 10.1038/s41598-017-06716-9 28740247 PMC5524752

[B49] Yeung Y. W. S. Ma Y. Liu S. Y. Pun W. H. Chua S. L. (2022). Prevalence of alcohol-tolerant and antibiotic-resistant bacterial pathogens on public hand sanitizer dispensers. J. Hosp. Infect. 127, 26–33. 10.1016/j.jhin.2022.05.017 35690267 PMC9176178

[B50] Zang Y. T. Bing S. Li Y. J. Shu D. Q. (2019). Application of slightly acidic electrolyzed water and ultraviolet light for *Salmonella enteritidis* decontamination of cell suspensions and surfaces of artificially inoculated plastic poultry transport coops and other facility surfaces. Poult. Sci. 98, 6445–6451. 10.3382/ps/pez520 31529076 PMC8913986

[B51] Zhao F. Wang S. Li Y. Wang J. Wang Y. Zhang C.. (2021). Surfactant cocamide monoethanolamide causes eye irritation by activating nociceptor TRPV1 channels. Br. J. Pharmacol. 178, 3448–3462. 10.1111/bph.15491 33837959 PMC11164132

[B52] Zheng Z. Huang Y. Liu L. Wang L. Tang J. (2023). Interaction between microplastic biofilm formation and antibiotics: effect of microplastic biofilm and its driving mechanisms on antibiotic resistance gene. J. Hazard. Mater. 459:132099. 10.1016/j.jhazmat.2023.132099 37517232

[B53] Zhu M. Yang Y. Wang M. Li X. Han R. Chen Q.. (2021). A deep insight into the suppression mechanism of *Sedum alfredii* root exudates on *Pseudomonas aeruginosa* based on quorum sensing. Ecotoxicol. Environ. Saf. 217:112240. 10.1016/j.ecoenv.2021.112240 33901783

