# Supplementary material for: Thymol combined with SAEW for the eradication of mature Pseudomonas aeruginosa biofilms and reduction of bacterial virulence
Source: Front Microbiol. 2025 Jun 27;16:1547632. doi: 10.3389/fmicb.2025.1547632 (PMC12245831; doi:10.3389/fmicb.2025.1547632)
Supplement: Supplementary file 1 [file Table_1.docx]

**Table S1. Primers used in this study**

| Target gene | Name | Sequence (5’ to 3’) |
| --- | --- | --- |
| *lasA* | lasA-F | CTACAGCATCAACCCGAAAG |
|  | lasA-R | TAGCGCCGCGACAACT |
| *lasB* | lasB-F | GTTCTATCCGCTGGTGTCG |
|  | lasB-R | CGCTGCCCTTCTTGATG |
| *rhlA* | rhlA-F | GGCGATCGGCCATCT |
|  | rhlA-R | AGCGAAGCCATGTGCTGAT |
| *pqsA* | pqsA-F | GACCGGCTGTATTCGATTC |
|  | pqsA-R | GCTGAACCAGGGAAAGAAC |
| *pqsE* | pqsE-F | GATGATGACCTGTGCCTGTT |
|  | pqsE-R | CGCCCAAACCAATTCGGCAT |
